# A curated transcriptome dataset collection to investigate the development and differentiation of the human placenta and its associated pathologies

**DOI:** 10.12688/f1000research.8210.2

**Published:** 2016-05-11

**Authors:** Alexandra K. Marr, Sabri Boughorbel, Scott Presnell, Charlie Quinn, Damien Chaussabel, Tomoshige Kino

**Affiliations:** 1 Sidra Medical and Research Center, Doha, Qatar; 2 Systems Immunology Division, Benaroya Research Institute, Seattle, WA, USA

**Keywords:** Transcriptomics, Bioinformatics, Placenta, Trophoblast, Diabetes, Pre-eclampsia, IUGR, trophoblast differentiation

## Abstract

Compendia of large-scale datasets made available in public repositories provide a precious opportunity to discover new biomedical phenomena and to fill gaps in our current knowledge. In order to foster novel insights it is necessary to ensure that these data are made readily accessible to research investigators in an interpretable format. Here we make a curated, public, collection of transcriptome datasets relevant to human placenta biology available for further analysis and interpretation via an interactive data browsing interface. We identified and retrieved a total of 24 datasets encompassing 759 transcriptome profiles associated with the development of the human placenta and associated pathologies from the NCBI Gene Expression Omnibus (GEO) and present them in a custom web-based application designed for interactive query and visualization of integrated large-scale datasets ( http://placentalendocrinology.gxbsidra.org/dm3/landing.gsp). We also performed quality control checks using relevant biological markers. Multiple sample groupings and rank lists were subsequently created to facilitate data query and interpretation. Via this interface, users can create web-links to customized graphical views which may be inserted into manuscripts for further dissemination, or e-mailed to collaborators for discussion. The tool also enables users to browse a single gene across different projects, providing a mechanism for  developing new perspectives on the role of a molecule of interest across multiple biological states. The dataset collection we created here is available at: http://placentalendocrinology.gxbsidra.org/dm3.

## Introduction

We aimed to make available via an interactive web-based application a collection of transcriptome datasets curated from the GEO public repository for their relevance to human placental development and pathology.

The placenta is a fetal organ indispensable for the establishment and maintenance of pregnancy. It connects the fetus to the maternal uterine wall via the umbilical cord, supplies nutrients and oxygen to the fetus, allows elimination of fetal waste, protects it from maternal infections and produces various hormones required for maintaining pregnancy ^1^. The placental trophoblasts play critical roles for all of these activities by acting as an interface between fetus and mother ^2^. In early embryogenesis, trophoblasts are the first cells differentiating from the fertilized egg, and eventually fusing with each other: a process transforming the monolayer cytotrophoblasts into syncytiotrophoblasts ^3^. Several morphological and biochemical changes occur during this fusion process throughout pregnancy (known as trophoblast differentiation) ^4^ to guarantee the development and appropriate functionality of the placenta ^1^. Failure in placental formation, differentiation and function, particularly by trophoblast dysfunction, impacts fetal development and is associated with a wide range of pathologies, including gestational diabetes, hypertension, pre-eclampsia and intrauterine growth restriction (IUGR) of the fetus ^5^. In addition, exposure of placenta to toxic compounds by a mother’s cigarette smoking alters the transcriptome of placental and fetal cells ^6^. Indeed, maternal tobacco use causes various pregnancy-associated problems, including spontaneous fetal abortion, placental abruption, preterm birth, stillbirth, fetal growth restriction, and sudden infant death syndrome ^6, 7^.

With over 65,000 data deposited into the NCBI Gene Expression Omnibus (GEO), a public repository for functional genomics data, it can be challenging to identify datasets relevant to a particular research area. Indeed, GEO was primarily designed for the purpose of data archiving rather than data browsing and downstream analysis. Thus, we identified datasets from GEO particularly relevant to our interest in the development and pathology of the human placenta and uploaded them to the custom web-based Gene Expression Browser application (GXB) ( http://placentalendocrinology.gxbsidra.org/dm3/landing.gsp), which provides seamless access to the data. GXB allows browsing and interactive visualization of large volumes of heterogeneous data. It also provides access to rich contextual information essential for data interpretation, such as: detailed gene information, relevant literature, study design, and sample information. In addition, the user can customize data plots by adding multiple layers of parameters, modify the sample order and generate links that can be shared via e-mail or used in future publications.

Thus we provide a resource enabling browsing of datasets relevant to placental development and pathology that offers a unique opportunity to identify genes that play a role in placental development/differentiation and are commonly modulated in pregnancy-associated diseases.

## Material and methods

111 datasets, potentially relevant to the development and pathology of the human placenta, were identified in GEO using the following search query: “Homo sapiens[Organism] AND placenta[DESC]”. The majority of retrieved datasets were generated using Illumina or Affymetrix microarrays besides a few other commercial platforms.

The relevance of each one of the entries retreived with our query pertaining to “development and pathology of the human placenta” was assessed on a case-by-case basis. This process included reading the NCBI’s GEO-linked article and examining the list of samples profiled and their annotations as well as the study design. We selected for studies using primary placenta cells, placenta tissue or trophoblast cell lines comparing a) healthy with diseased samples or smoker vs. non-smoker or b) placenta cells in different differentiation/gestational stages. Finally we retrieved 24 curated datasets encompassing 759 transcriptome profiles, including 22 datasets generated using primary placenta cells and 2 datasets generated using trophoblast cell lines. Among the 24 datasets, there are 18 studies comparing control vs. diseased/smoker placenta cells, and 6 investigating transcriptomic changes during placental development and/or trophoblast differentiation. Among the many noteworthy datasets, several stood out, such as an extensive study comparing the diseased placentas with pre-eclampsia or unexplained IUGR vs. normal placentas (GSE24129) ^8^. The datasets that comprise our collection are listed in Table 1.

**Table 1.** List of all datasets included in our curated collection, also available at http://placentalendocrinology.gxbsidra.org/dm3. For more information, see http://placentalendocrinology.gxbsidra.org/dm3/geneBrowser/list: PLAC1, placenta specific 1: CSH1, placental lactogen: XIST, X-inactive specific transcript NP: not published, no PubMed publication for this data set.

Title	Platform	Number of Samples	validation	GEO ID	Ref.
Altered Gene Expression Profile of Microvascular Endothelium in Placentas from IUGR/Preeclamptic Pregnancies	Affymetrix Human Genome U133 Plus 2.0 Array	10	PLAC1, CSH1, XIST	**GSE25861**	12
Chorionic villus sampling (CVS) microarray in preeclampsia	Affymetrix Human Genome U133 Plus 2.0 Array	12	PLAC1, CSH1	**GSE12767**	13
Comprehensive Study of Tobacco Smoke-Related Transcriptome Alterations in Maternal and Fetal Cells	Illumina HumanRef-8 v3.0 expression beadchip	183	PLAC1, CSH1	**GSE27272**	6
Culturing Cytotrophoblasts Reverses Gene Dysregulation in Preeclampsia Revealing Possible Causes	Affymetrix Human Genome U133 Plus 2.0 Array	39	PLAC1, CSH1	**GSE40182**	14
Deregulation of Gene Expression induced by Environmental Tobacco Smoke Exposure in Pregnancy	Illumina HumanRef-8 v3.0 expression beadchip	104	PLAC1, CSH1	**GSE30032**	15
Differential gene expression in Trophoblast cell cultures	Affymetrix Human Genome U133 Plus 2.0 Array	2	PLAC1, CSH1	**GSE4100**	NP
Differentially expressed microRNAs revealed by molecular signatures of Preeclampsia and IUGR in human placenta	Illumina human-6 v2.0 expression beadchip	94	PLAC1, CSH1, XIST	**GSE35574**	16
Dysregulation of the circulating and tissue-based renin- angiotensin system in preeclampsia	Affymetrix Human Genome U133 Plus 2.0 Array	6	PLAC1, CSH1	**GSE6573**	17
Full-term placenta, smokers and non-smokers	Affymetrix Human Genome U133 Plus 2.0 Array	10	PLAC1, CSH1, XIST	**GSE7434**	18
Gene expression profiling for placentas from pre- eclamptic, unexplained FGR and normal pregnancies.	Affymetrix Human Gene 1.0 ST Array	24	PLAC1, CSH1	**GSE24129**	8
Gene expression profiling indicates inflammatory pathways involved in IUGR due to placental insufficiency	ABI Human Genome Survey Microarray Version 2	16	PLAC1	**GSE12216**	19
Gene expression profiling of trophoblast cells	Affymetrix Human Genome U133A Array	11	PLAC1, CSH1	**GSE9773**	19
Genome wide analysis of placental malaria	Affymetrix Human Genome U133 Plus 2.0 Array	20	PLAC1, CSH1, XIST	**GSE7586**	20
Global placental gene expression profiling in the first and third trimesters of normal human pregnancy	ABI Human Genome Survey Microarray Version 2	37	PLAC1	**GSE28551**	4
Hypoxia induced HIF-1/HIF-2 activity alters trophoblast transcriptional regulation and promotes invasion	Illumina HumanHT-12 V4.0 expression beadchip	18	PLAC1, CSH1	**GSE65271**	21
Increased placental expression and maternal serum levels of apoptosis-inducing TRAIL in recurrent miscarriage	Affymetrix Human Genome U133 Plus 2.0 Array	10	PLAC1, CSH1	**GSE22490**	21
Mid-gestational gene expression profile in placenta and link to pregnancy complications	Affymetrix Human Genome U133 Plus 2.0 Array	4	PLAC1, CSH1	**GSE37901**	22
Placental gene expression in severe preeclampsia.	ABI Human Genome Survey Microarray Version 2	43	PLAC1	**GSE10588**	22
Profiling Gene Expression in Human Placentae of Different Gestational Ages: an OPRU Network and UW SCOR Study	Affymetrix Human Genome U133 Plus 2.0 Array	12	PLAC1, CSH1	**GSE9984**	23
Transcriptomic profiling of human placental trophoblasts in response to Enterococcus faecalis invasion	Illumina HumanHT-12 V4.0 expression beadchip	4	PLAC1, CSH1, XIST	**GSE75626**	23
Genome-wide analysis of gene expression in placentas derived from patients with preeclampsia	Illumina HumanHT-12 V4.0 expression beadchip	12	PLAC1, CSH1	**GSE30186**	24
Genomic expression profiles of blood and placenta in Chinese women with gestational diabetes	Aalborg University Illumina human-6 v2.0 expression beadchip	5	PLAC1, CSH1	**GSE19649**	NP
Severe Preeclampsia-Related Changes in Gene Expression at the Maternal-Fetal Interface Include Siglec-6 and Pappalysin-2	Affymetrix Human Genome U133A Array	23	PLAC1, CSH1	**GSE14722**	25
Transcriptional Profiling of Human Placentas from Pregnancies Complicated by Preeclampsia Reveals Disregulation of Sialic Acid Acetylesterase and Immune Signaling Pathways	Illumina human-6 v2.0 expression beadchip	60	PLAC1, CSH1	**GSE25906**	26

Once a final selection had been made, each dataset was downloaded from GEO in the SOFT file format. These files were then uploaded to the “placentaendocrinology” instance of GXB ( http://placentalendocrinology.gxbsidra.org/dm3), an interactive web-based application developed at the Benaroya Research Institute, hosted on the Amazon Web Services cloud ^9^. Information about samples and study design was also uploaded. Samples were then grouped based on relevant study variables and genes were ranked based on specified group comparisons. Details of the GXB software are described in a recent publication ^9^. A tutorial for this software is also available online ( https://gxb.benaroyaresearch.org/dm3/tutorials.gsp#gxbtut). GXB provides the user with a means to navigate and filter the dataset collection available at ( http://placentalendocrinology.gxbsidra.org/dm3). Briefly, the datasets of interest can be quickly identified either by filtering with the pre-existing criteria listed in the column located in the left side of the dataset navigation window or by entering a query term in the search box located at the top of the window. Clicking one of the studies listed in the dataset navigation window opens a viewer, which is designed to support interactive browsing and graphic representations of the data in an interpretable format. This interface was developed to navigate ranked gene lists and to display expression results in a figure with a context-rich environment. Selecting a gene from the rank-ordered list located on the left side of the navigation window displays its expression values on the interactive plot. The drop-down menus located directly above the graphical display provide the user the following functions: a) Exporting the created graph as a portable network graphics (png) image or a csv file for performing a separate analysis. b) Changing ranking of genes in the list; this function allows the user to manipulate the ways of ranking the genes depending on his/her interest, or to include only the genes selected based on specific biological interest. c) Changing grouping of the samples (by using “Group Set” button); for example, the user can convert the groups created based on “cell type” to those on “disease type”. d) Identifying individual samples within a group using categorical or continuous variables (e.g., mode of delivery, ethnic group and age). e) Toggling between the bar chart view and a box plot view, with demonstration of the values as a single plot for each sample. Samples are split into different categories or groups whatever they are displayed in a bar chart or box plot. f) Providing a color legend for sample groups. g) Providing a color legend for the categorical information overlaid at the bottom of the graph. h) Selection of categorical information that is to be overlaid at the bottom of the graph. Using this function, the user can display, for example, gender or smoking status, at the bottom of the figure. The data without contextual information have no intrinsic utility. It is therefore important to capture and display the context information in the graph, so that the viewer can interpret the data shown in the graph. GXB provides functions for organizing the context information in the tabs located just above the graphical display. The tabs can be hidden to make more room for graphs, or be reappeared by clicking the blue “Show Info Panel” button located on the top right corner of the graphical display. Information on the genes selected from the list located in the left side of the graphical display is shown under the “Gene” tab. Information on the study for the demonstrated dataset is available under the “Study” tab. Detailed information of individual samples is provided under the “Sample” tab. Rolling the mouse cursor over a sample in either the bar chart or box plot view while displaying the “Sample” tab displays any clinical, demographic, or laboratory information available for that sample. Finally, the “Downloads” tab allows the advanced users to retrieve the original data for further analysis using other softwares/tools. It also provides all available sample annotation data along with the expression data. Other functionalities are provided under the “Tools” drop-down menu located in the top right corner of the user interface. Some of the notable functionalities available through this menu include: a) Annotations, which provides access to all the ancillary information about the study, samples and dataset organized across different tabs; b) Cross project view, which provides the ability to browse through all available studies while focusing on a the currently selected gene; c) Copy link, which generates a mini-URL encapsulating information about the display settings in use and that can be saved and shared with others (clicking on the envelope icon on the toolbar inserts the url in an email message via the local email client); d) Chart options, which gives user the option to customize chart labels.

## Dataset validation

We performed quality control checks on the datasets loaded on GXB by validating the expression of placental marker genes. Specifically, we used placenta specific 1 (PLAC1) ^10^ and human placental lactogen (CSH1) ^11^ ( Table 1). As expected, all samples from the datasets we incorporated in our collection expressed these two placental markers (hyperlinked for easy access in Table 1), except some samples in GSE12216, GSE28551 and GSE10588, where CSH1 was not measured.

Gender specific expression of the XIST transcript (in the samples for which sex information was available) was also examined to determine its concordance with demographic information provided with the GEO datasets ( Table 1). An overall accordance of a high XIST expression in female samples compared to a low XIST expression in male samples was determined in our entire dataset collection ( Table 1). Based on our experience, concordance should be close to 100%. Levels of concordance closer to 50%, which were not observed here, would indicate a potential error with handling of samples during processing that may dramatically affect data analysis and interpretation (e.g. plate inversion).

**Figure 1.**
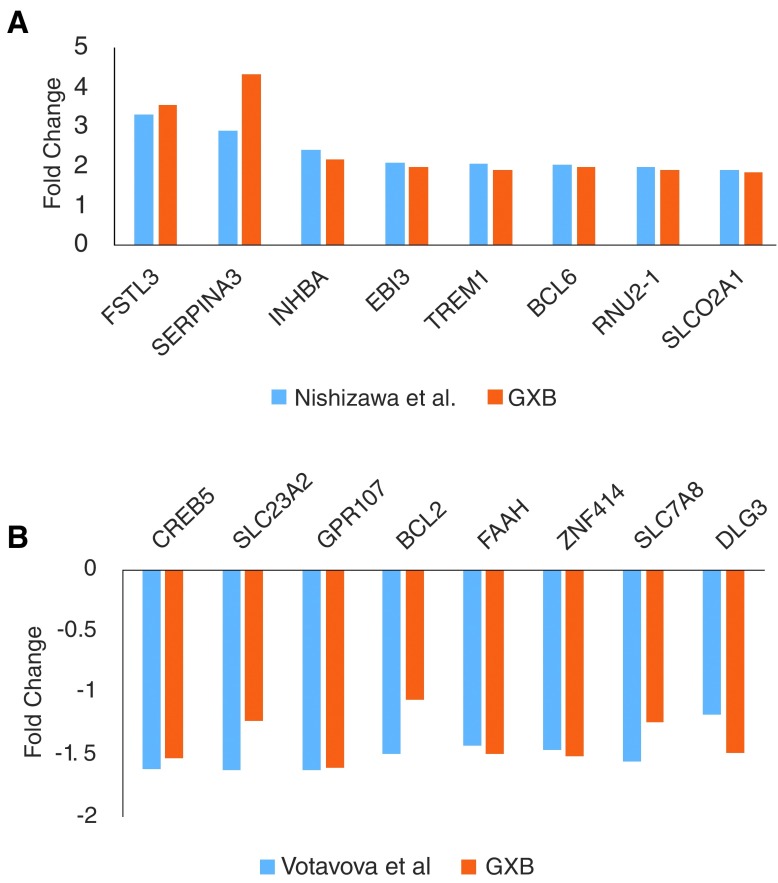
Verification of differential gene expression (Fold Changes) in GXB compared to the values published in the originating manuscript. Two datasets were selected for validation of differential expression: GSE24129 ( **A**) and GSE 30032 ( **B**). The first 8 genes were selected from Table 2 in Nishizawa *et al.* (for GSE24129) and from supplemental material Table in Votavova *et al.* (for GSE30032).

We also verified and compared the differential expression using the Fold Change values obtained from GXB or the original manuscript. For this verification process, two datasets were selected GSE24129 and GSE30032. As shown in Figure 1, Fold Change values obtained from GXB correlate with those obtained from the original published article.

## Data availability

The data referenced by this article are under copyright with the following copyright statement: Copyright: © 2016 Marr AK et al.

All datasets included in our curated collection are available publically at the NCBI GEO website: http://www.ncbi.nlm.nih.gov/gds/. They were cited in our manuscript along with their GEO accession numbers (e.g. GSE24129). Signal files and sample description files for each uploaded GEO dataset can also be downloaded from the GXB tool under the “downloads” tab after accessing our dataset collection.
