# Supplementary material for: A curated transcriptome dataset collection to investigate the development and differentiation of the human placenta and its associated pathologies
Source: F1000Res. 2016 May 11;5:305. Originally published 2016 Mar 9. [Version 2] doi: 10.12688/f1000research.8210.2 (PMC4897750; doi:10.12688/f1000research.8210.2)
Supplement: Peer review history

# Peer review: Referee response for version 2

**DOI:** 10.5256/f1000research.9407.r14210

**Published:** 2016-06-07

**Authors:** Brian Cox

**Affiliations:** 1 Department of Physiology, Faculty of Medicine, University of Toronto, Toronto, ON, Canada

I find the authors responses thoughtful and encouraging that this may become a stable resource that will expand.

I have read this submission. I believe that I have an appropriate level of expertise to confirm that it is of an acceptable scientific standard.

# Peer review: Referee response for version 1

**DOI:** 10.5256/f1000research.8830.r12946

**Published:** 2016-04-06

**Authors:** Maria Belen Rabaglino

**Affiliations:** 1 Center of Excellence in Products and Processes of Cordoba, National Scientific and Technical Research Council, Cordoba, Argentina

The authors of this article have collected several gene expression datasets, related with the development of the human placenta and associated pathologies, from the public database Gene Expression Omnibus (GEO) to analyze them in the context of a web application known as Gene Expression Browser (GXB) platform.

This web tool provides a graphical interface to search and visualize gene status across different datasets. The collection of datasets presented on this article makes this platform particularly useful to gather information about transcriptome profiles related to human placenta and associated pathologies. Undoubtedly, this tool allows fast searching of a gene of interest on those datasets, which could facilitate visualization of results from the corresponding studies and helps in manuscripts or grants writing.

There is no information on this manuscript about the statistical analysis followed to rank the genes on each dataset but it refers to the publication that details the GXB software ^1^. Here, it is specified that genes are ranked according their differential expression using the limma package, and the links to the R scripts are showed. However, the application should include the information about the normalization/background correction methods used on each dataset. Different methods could yield different expression measures ^2^ and so modify the gene ranking.

Also, comparisons of gene expression across studies could be a powerful feature of this program, but for that is necessary to apply a method to remove batch effects.

Another suggestion is to extend the verification of the datasets including genes that are known to be deregulated in placental pathologies --such as LEP up-regulation in preeclamptic placenta ^3^^,^^4^--. An explanation about these genes along with graphs obtained from the web application could be useful for the reader as an example of its utility.

Finally, the application should include the date of the last update. Obviously, new placenta-related datasets might be uploaded into the GEO database and so they should be added to the collection.

I have read this submission. I believe that I have an appropriate level of expertise to confirm that it is of an acceptable scientific standard.

## References

[1] SpeakeCPresnellSDomicoKZeitnerBBjorkAAndersonDMasonMJWhalenEVargasOPopovDRinchaiDJourde-ChicheNChicheLQuinnCChaussabelD: An interactive web application for the dissemination of human systems immunology data. *J Transl Med*.2015;13: 10.1186/s12967-015-0541-x 196 10.1186/s12967-015-0541-x 26088622PMC4474328

[2] IrizarryRAWuZJaffeeHA: Comparison of Affymetrix GeneChip expression measures. *Bioinformatics*.2006;22(7) : 10.1093/bioinformatics/btk046 789-94 10.1093/bioinformatics/btk046 16410320

[3] TejeraEBernardesJRebeloI: Co-expression network analysis and genetic algorithms for gene prioritization in preeclampsia. *BMC Med Genomics*.2013;6: 10.1186/1755-8794-6-51 51 10.1186/1755-8794-6-51 24219996PMC3829810

[4] KaartokallioTCerveraAKyllönenALaivuoriK: Gene expression profiling of pre-eclamptic placentae by RNA sequencing. *Sci Rep*.2015;5: 10.1038/srep14107 14107 10.1038/srep14107 26388242PMC4585671

# Peer review: Referee response for version 1

**DOI:** 10.5256/f1000research.8830.r12824

**Published:** 2016-03-23

**Authors:** Brian Cox

**Affiliations:** 1 Department of Physiology, Faculty of Medicine, University of Toronto, Toronto, ON, Canada

The web based utility is well assembled and easy to navigate and have very nice layout and graphic capabilities. A large spectrum of RNA microarray human placental sample sets have been assembled. 

Reservations are:

The verification of the datasets using CSH1 and PLAC1 is rather minimal. Can you verify the differential expression observed in the originating manuscripts? At least in a couple of examples, such as one smoking and one preeclampsia/IUGR. Figures with examples graphics from these validations could be helpful. 

In many sample sets there exist batch effects. In some cases the depositors have identified the batches, but have not corrected for these, in other cases batches can be observed even when not formally identified by the depositor. For maximum utility these effects should be removed or a tool supplied to identify and remove them.

Further to the need for batch correction, viewing a gene across multiple uncorrected/non-normalized data sets is not very informative.

The web utility was frequently unavailable during the review process. A more stable platform or redundant server system should be established to limit the down time of this application. What user load can the server handle, how many concurrent users can the server handle?

Only transcription arrays are used in the assembled data sets, there are numerous data sets involving DNA methylation, microRNAs and protein. Incorporation of these data sets would create a true systems level database of the human placenta.

Improved utility would come from enabling merging of multiple datasets to perform meta analysis of aggregate analysis. This would require the installation of a batch corrections scheme or method/s. 

There is also a growing resource of sequencing based data sets that have been neglected in this application/manuscript. Once converted into a table of gene expression/counts, it should not be too challenging to incorporate these datasets as well. 

User training could be improved with additional figures to support descriptions of the utility and data sets. Better would be a vignette containing worked examples of the different types of analyses to generate the table and figures discussed in the paper. Similar worked vignette examples are frequently found in R libraries.

I have read this submission. I believe that I have an appropriate level of expertise to confirm that it is of an acceptable scientific standard, however I have significant reservations, as outlined above.

# Response

**Published:** 2016-05-02

**Authors:** Alexandra Marr

**Affiliations:** Sidra Medical and Research Center, Qatar

*The web based utility is well assembled and easy to navigate and have very nice layout and graphic capabilities. A large spectrum of RNA microarray human placental sample sets have been assembled.* 

**Reservations are:**

*1. The verification of the datasets using CSH1 and PLAC1 is rather minimal. Can you verify the differential expression observed in the originating manuscripts? At least in a couple of examples, such as one smoking and one preeclampsia/IUGR. Figures with examples graphics from these validations could be helpful*. 

We included a new figure (new Figure 1) showing the verification of differential gene expression in GXB compared to the values published in the originating manuscript. We also included the following paragraph in the “Dataset Validation” section of the manuscript: “We also verified and compared the differential expression using the Fold Change values obtained from GXB or the original manuscript. For this verification process, two datasets were selected GSE24129 and GSE30032. As shown in Figure 1, Fold Change values obtained from GXB correlate with those obtained from the original published article.”

*2. In many sample sets there exist batch effects. In some cases the depositors have identified the batches, but have not corrected for these, in other cases batches can be observed even when not formally identified by the depositor. For maximum utility these effects should be removed or a tool supplied to identify and remove them.*

*Further to the need for batch correction, viewing a gene across multiple uncorrected/non-normalized data sets is not very informative.*

The reviewer raises an important point. Batch effects resulting in non-biological experimental variation across multiple batches of microarray experiments have been well documented. Several methods are available to correct for batch effects, such as Combat in ‘R’ ( http://www.r-project.org/). In the context of a dataset compendium batch effects should be considered at two different levels:

a) Within datasets: Unfortunately batch-processing information is available for only a small minority of datasets deposited in GEO (6% in the case of our “placental endocrinology” dataset collection), which makes systematically correcting for such effects impossible. This limitation of course affects any investigator inclined to use GEO as a resource. The practice of randomizing groups across runs, which avoids having batch effects confounding the analysis, is fortunately widely accepted. But it cannot be altogether discounted.

However, this is where one of the distinct advantage of working with a compendium of dataset comes into play: a conclusion regarding differential expression of a given gene can be based on observation of such differences in not just one but multiple datasets, obtained independently by different investigators, in different parts of the world, using different array platforms. The conclusions that can be thus derived are especially robust. This point is illustrated by one of our recent contribution to F1000Research: http://f1000research.com/articles/4-89/v1

b) Across datasets: We have previously implemented strategies for meta-analyses per-se, focusing on blood transcriptome data, and found it necessary to reduce the information from each dataset to p-values (comparison within each dataset of cases to a control group; 17724127, 16461797) or co-clustering information (module repertoire analyses 24662387). While GXB is primarily designed as a data browser it is possible to employ it similarly (but with added flexibility) for “meta-interpretation” (rather than meta-analysis). This consists in interpreting findings for a given gene across tens or hundreds of datasets, thus affording a unique perspective to the investigators and an effective means to identify and address knowledge gaps. This again is illustrated in the article mentioned above: http://f1000research.com/articles/4-89/v1. Notably, the cross-project view functionality also provides an independent search for datasets with genes that have a certain fold-changes above a chosen threshold.

*3. The web utility was frequently unavailable during the review process. A more stable platform or redundant server system should be established to limit the down time of this application. What user load can the server handle, how many concurrent users can the server handle?*

We thank the reviewer for this critical feedback. Our instance was created on the Amazon Elastic Compute Cloud (Amazon Web services). The downtime was not due to the GXB application per se but rather to some missing packages related to the tomcat server. These packages have been fixed and the downtime issue is now resolved. The number of concurrent users is configurable and it is related to tomcat web server. The current configuration is 200 concurrent users. This can be reconfigured as needed.

*4. Only transcription arrays are used in the assembled data sets, there are numerous data sets involving DNA methylation, microRNAs and protein. Incorporation of these data sets would create a true systems level database of the human placenta.*

These are great ideas. We are currently working on the interactive web application (GXB) to be able to expand to other platforms such as working on SomaLogic data etc. We do see the establishment of our instance as an ongoing project, where additional datasets can be uploaded on demand (see “Request to load a Sample Set” function on the upper right corner of the web application page < http://placentalendocrinology.gxbsidra.org/dm3/geneBrowser/list>), for example when new datasets are published and deposited, and according to advances in our GXB technology.

*5. Improved utility would come from enabling merging of multiple datasets to perform meta analysis of aggregate analysis. This would require the installation of a batch corrections scheme or method/s.*

We agree with the reviewer’s point of view. However, it is a difficult problem to address at the gene level. We are currently working on approaches and tools involving those modular repertoires (please see Chaussabel D. *et al*. 2008 Immunology 29:150-64 and Chaussabel D. & Baldwin N. 2014 Nat Rev Immunol. 14:271-80). See also our comments above pertaining to the point raised earlier regarding batch correction.

*6. There is also a growing resource of sequencing based data sets that have been neglected in this application/manuscript. Once converted into a table of gene expression/counts, it should not be too challenging to incorporate these datasets as well.*

We agree that it would be very valuable to include RNAseq datasets in the future. We are not at this time in measure to accommodate this data type routinely but as a proof of principle a trial RNA-seq dataset have been setup to display RNA-seq profiles of immune cell subsets isolated from subjects with a wide range of diseases (GSE60424, https://gxb.benaroyaresearch.org/dm3/geneBrowser/show/396, http://journals.plos.org/plosone/article?id=10.1371/journal.pone.0109760).

*7. User training could be improved with additional figures to support descriptions of the utility and data sets. Better would be a vignette containing worked examples of the different types of analyses to generate the table and figures discussed in the paper. Similar worked vignette examples are frequently found in R libraries.*

Indeed, thank you for suggesting this. We started working on a ‘User Training’ document with detailed, step-by-step descriptions. This document will be published in the Collective Data Access channel of F1000Research very soon.
